# Text-Book of Medicine

**Published:** 1894-01

**Authors:** 


A TEXT BOOK OF MEDICINE, for Students and Practitioners, by Dr. Adolf Strümpell, Professor and Director of the Medical Clinique at Erlangen. Translated, by permission, from the sixth German edition, by Herman F. Vickery, A. B., M. D., Instructor in Clinical Medicine, Harvard Medical School, etc., and Philip Coombs Knapp, A. M., M. D., Physician to Out-patients with Diseases of the Nervous System, Boston City Hospital, etc. With Editorial Notes by Frederick C. Shattuck, A. M., M. D., Jackson Professor of Clinical Medicine, Harvard Medical School, etc. Second American edition. With 111 Illustrations. 8 vo. 1043 pages. Cloth, $6; sheep, $7. D. Appleton & Co., publishers, 1, 3 and 5 Bond Street, New York.

Strümpell's Text Book of Medicine has been a favorite with American physicians since the first appearance of the American edition. It has received the high compliment of being adopted and recommended as a text book by the majority of the medical schools of the United States. Many important changes have been made and much new matter added in making up this, the second American edition, and there can be but little doubt that it will now increase in popularity more rapidly than ever before.

Dr. Strümpel is recognized as one of the ablest physicians and teachers in Europe, and the translators rank high in the medical profession of the United States.

One special feature in which this work surpasses most of the text books translated from a foreign language into our own, and for which the translators deserve especial commendation, is the consideration of a number of diseases largely prevalent in the

United States, but almost unknown to Germany. The translators, realizing the necessity of making the book conform to the wants of the American student and practitioner, have supplemented the original German work with the consideration of such diseases as are of interest to Americans. They have also discussed many subjects from an American standpoint, independent of the views of the author. We thus get the views of leading German and American physicians on a variety of subjects. A very large part of the book (more than 300 pages) is devoted to the consideration of diseases of the nervous system. This is quite a change from the text book of a few years since, and the majority of the profession of this country will appreciate the full discussion of this class of diseases about which so much remains yet to be learned.

The mechanical work of the book is good, though the print is rather smaller than usual in the leading text books. This, however, was necessary that the book might be of a convenient size. H.

